# A Sedentary and Unhealthy Lifestyle Fuels Chronic Disease Progression by Changing Interstitial Cell Behaviour: A Network Analysis

**DOI:** 10.3389/fphys.2022.904107

**Published:** 2022-07-08

**Authors:** Patricia Huston

**Affiliations:** ^1^ Department of Family Medicine, Faculty of Medicine, University of Ottawa, Ottawa, ON, Canada; ^2^ Institut du Savoir Montfort (Research), University of Ottawa, Ottawa, ON, Canada

**Keywords:** chronic disease, sedentariness, autonomic nervous system, physical activity, microenvironment, macrophages (M1/M2), chronic low-grade inflammation, fibroblasts

## Abstract

Managing chronic diseases, such as heart disease, stroke, diabetes, chronic lung disease and Alzheimer’s disease, account for a large proportion of health care spending, yet they remain in the top causes of premature mortality and are preventable. It is currently accepted that an unhealthy lifestyle fosters a state of chronic low-grade inflammation that is linked to chronic disease progression. Although this is known to be related to inflammatory cytokines, how an unhealthy lifestyle causes cytokine release and how that in turn leads to chronic disease progression are not well known. This article presents a theory that an unhealthy lifestyle fosters chronic disease by changing interstitial cell behavior and is supported by a six-level hierarchical network analysis. The top three networks include the macroenvironment, social and cultural factors, and lifestyle itself. The fourth network includes the immune, autonomic and neuroendocrine systems and how they interact with lifestyle factors and with each other. The fifth network identifies the effects these systems have on the microenvironment and two types of interstitial cells: macrophages and fibroblasts. Depending on their behaviour, these cells can either help maintain and restore normal function or foster chronic disease progression. When macrophages and fibroblasts dysregulate, it leads to chronic low-grade inflammation, fibrosis, and eventually damage to parenchymal (organ-specific) cells. The sixth network considers how macrophages change phenotype. Thus, a pathway is identified through this hierarchical network to reveal how external factors and lifestyle affect interstitial cell behaviour. This theory can be tested and it needs to be tested because, if correct, it has profound implications. Not only does this theory explain how chronic low-grade inflammation causes chronic disease progression, it also provides insight into salutogenesis, or the process by which health is maintained and restored. Understanding low-grade inflammation as a stalled healing process offers a new strategy for chronic disease management. Rather than treating each chronic disease separately by a focus on parenchymal pathology, a salutogenic strategy of optimizing interstitial health could prevent and mitigate multiple chronic diseases simultaneously.

## Introduction

Prior to the COVID-19 pandemic, Americans spent almost $4 trillion a year on healthcare services, 90% of which was spent on chronic disease (Buttorff et al., 2017; Martin et al., 2021). Sixty percent of all adults in the United States have at least one chronic condition, and 80% of older adults have multiple chronic diseases (Buttorff et al., 2017). Yet despite all the medications and all the health care given, many chronic conditions—such as heart disease, stroke, diabetes, chronic lung disease, and Alzheimer’s disease—are in the top ten causes of death and have remained there largely unchanged for decades (Kochanek et al., 2019). It has been estimated that this “silent epidemic” of chronic disease killed almost four times more Americans in 2020 than COVID-19 (Ahmad and Anderson, 2021; Rippe, 2021).

The World Health Organization (WHO) reported that, between 2000–2019, the world’s most common cause of death was ischemic heart disease, and it was responsible for the largest increase in deaths worldwide over that time period (World Health Organization, 2020). And it is preventable. Multiple studies have shown a healthy lifestyle can help prevent myocardial infarctions regardless of genetic susceptibility (Abraham et al., 2016; Khera et al., 2016; Sotos-Prieto et al., 2016; Said et al., 2018). A *New England Journal of Medicine* study analyzed over 50,000 people and found that for those at high genetic risk, not smoking, avoiding obesity, regular physical activity, and a healthy diet were associated with a nearly 50% lower relative risk of coronary artery disease than those at high genetic risk with an unfavorable lifestyle (Khera et al., 2016). Another study of over 300,000 people found the effect size even larger (Said et al., 2018).

Physical activity is the cornerstone of a healthy lifestyle. A recent Cochrane review of 187 randomized controlled trials (RCTs) confirmed that both healthy and medically compromised individuals can significantly improve function, physical and mental health, and decrease mortality by exercising more (Posadzki et al., 2020). A clear dose-response relationship between physical activity and longevity has been found (Ekelund et al., 2019; Geidl et al., 2020). In contrast, WHO has noted that sedentariness—or insufficient physical activity—is one of the leading risk factors for premature mortality (World Health Organization, 2022). The general public knows that getting regular physical activity is good for you and unhealthy eating and stress are not, yet most Americans do not meet the United States guidelines for healthy eating or physical activity (Physical Activity Guidelines Advisory Committee, 2018), and anxiety levels and social unease are more common than ever.

Over the last two decades, research from multiple sources has shown that chronic disease is associated with chronic low-grade inflammation linked with an unhealthy lifestyle and aging (Fülöp et al., 2019). Low grade inflammation is a prominent feature of atherosclerosis (Wolf and Ley, 2019; Vergallo and Crea, 2020), cardiovascular disease (Fioranelli et al., 2018) and stroke (Murray et al., 2013). It plays a critical role in chronic lung disease (Yamasaki and Eeden, 2018), obesity (Kohlgruber and Lynch, 2015) and type 2 diabetes (Shapouri-Moghaddam et al., 2018), as well as cognitive impairment (Bettcher and Kramer, 2013) and Alzheimer’s disease (Giunta et al., 2008).

Two basic questions remain: *How* does an unhealthy lifestyle cause chronic low-grade inflammation? And: *How* does inflammation cause chronic disease progression? These questions are starting to be addressed by recent advances in physiology, such as understanding the interactions of the immune system with food and the microbiome, intercellular cross-talk, and the epigenetics of phenotypic shifts. And to bring this all together, is another relatively new field: network analysis.

Network analysis is the study of the interactions involved in complex physical, social and biological processes. It is often illustrated with a figure where key factors are represented by nodes and key influences and interactions among these factors are represented by edges or connecting lines. Nodes that have multiple interactions tend to have the most influence and are called hubs. Networks can be single, multiple, or multiple and hierarchical. Network analysis has been applied to physiology and medicine. Network physiology addresses how physiological systems and subsystems coordinate and interact to optimize health and adapt to changing physiologic states (Bartsch et al., 2015; Ivanov et al., 2016; Lehnertz et al., 2020). It has been applied to lifestyle factors, such as exercise, to examine the dynamic interactions of skeletal muscle with organs and fat (Balagué et al., 2020). And it has been applied more broadly to identify health as an emergent state that arises from hierarchical network interactions between a person’s external environment and their internal physiology (Sturmberg et al., 2019). Network medicine has been used to understand the interacting genetic and non-genetic factors associated with diabetes (Zhang et al., 2021) and to-analyze how genomic, proteomic, and metabolic factors lead to different disease phenotypes (Ivanov et al., 2016).

The aim of this network analysis is to consider how lifestyle is embedded in a network of influences, how the physical and social environment influences peoples’ lifestyles and how, in turn, both a healthy and an unhealthy lifestyle have an influence on chronic disease progression. This network analysis represents a map of the connections between the macroenvironment, an intermediary mesoenvironment, and the microenvironment. Evidence is presented that leads to the conclusion chronic disease progression arises from a stalled healing process orchestrated by immune and connective tissue cells in the microenvironment. When immune cells get dysregulated, it leads to chronic low-grade inflammation and when connective tissue cells get dysregulated it leads to progressive fibrosis. The outcome of this cellular behaviour is illustrated by describing what happens in the microenvironment with heart disease. Due to its scope, this analysis is not all-inclusive. Rather, a pathway of inter-connectivity is identified within and between these six networks to influence the very cells that either help to maintain and restore health or foster chronic disease progression.

## Two External Networks

**FIGURE 1:**
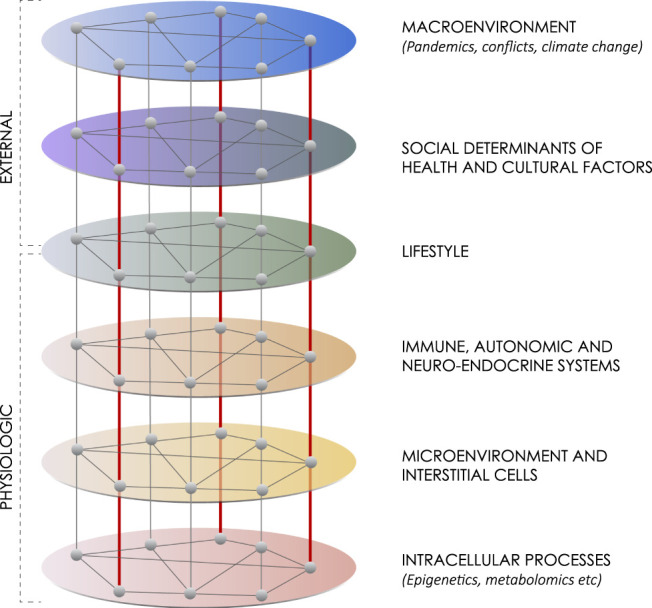
Lifestyle within a six-layer hierarchical network. This network diagram summarizes six layers of influences on maintaining health or advancing chronic disease. The first network includes macroenvironment influences like major weather, climate or political events, such as war. The second network includes social determinants of health and cultural factors. The third network includes lifestyle itself made up of physical activity, diet and stress or well-being. The fourth network includes the interacting immune, autonomic and neuroendocrine systems. The fifth network includes the interactions among interstitial cells within the microenvironment. The sixth network includes intracellular processes, such as epigenetics, genomics, transcriptomics and metabolomics. Although generally influences work from the top down, there can also be an emergence of influence from the bottom up.

A hierarchical network has distinct layers, that operate at different spatial and temporal scales. There are connections that occur among factors within a layer and between layers. Influences are generally top-down from one layer to the next, but it is possible to have emerging influences arise from the bottom up. As a result of this bi-directional potential, circular causality relationships among network levels can occur, creating self-regulating systems, such as negative feedback loops. These self-regulating systems can get disrupted, and either dysfunctional or spontaneous “new normal” causality relationships can develop as part of the body’s effort to adapt (Noble et al., 2019). In this network analysis there are six layers (Figure 1). Starting at the top there are two external networks that influence lifestyle.

### The Larger Macroenvironment

The two external networks that influence lifestyle are the larger macroenvironment and the local social and cultural environment. The larger macroenvironment is a network of interacting environmental and geopolitical factors. The COVID-19 pandemic is a stark example of this. By June 2022, there were over half a billion cases and over 6 million deaths reported worldwide (World Health Organization, 2022). Due to lockdowns in many parts of the world, even those who did not get ill, had been socially isolated, stressed and had become less active (Mukhtar, 2020). Other macroenvironmental factors including extreme weather events, such as flooding and heat waves, and political events such as war, all profoundly affect lifestyle and health (Rocque et al., 2021; Shahin et al., 2021).

### The Social and Cultural Environment

The second external network includes the social determinants of health and other cultural factors. When almost three in four American adults are overweight or obese (Fryar et al., 2020), this suggests more is at play than individual choice. Social determinants of health have identified that a healthy lifestyle is much easier to realize and maintain for those who have economic stability and access to healthy activities, health services and social support, than for those who do not (CDC, 2021). Although this is true, it is more complex than that (Frank et al., 2020). People’s lifestyle choices are profoundly shaped by their culture, family history and the social networks they are embedded in (Zhang et al., 2018), and this can trump the social determinants of health. For example, affluent executives may have all the social determinants of health but if they are constantly working and do not find time to eat a healthy diet or exercise, they are likely to develop a lifestyle-related chronic disease. The high prevalence of stress, screen-based activities and a diet high in fat and sugar in Western countries are part of Western culture. Fortunately, there are other culturally congruent influences for a healthy lifestyle, and this is the one level in the hierarchical network where personal choice can override countervailing influences.

## Lifestyle as a Network

Definitions of healthy and unhealthy lifestyles vary. Although there is general agreement that a healthy lifestyle includes regular physical activity and a healthy diet some definitions also include smoking cessation, social and psychological well-being, moderate to no alcohol use (World Health Organization, 1999) and adequate sleep (Banks and Dinges, 2007). Most descriptions of an unhealthy lifestyle include some marker of psycho-social stress, an unhealthy diet that typically leads to obesity, sedentariness (Mainous et al., 2010), and sometimes addictions (Weinstock et al., 2017). Whereas obesity, excess alcohol and addictions can be seen as signs of dysregulation (Koob and Volkow, 2016; Becker, 2017; Narayanaswami and Dwoskin, 2017), some aspects of a healthy lifestyle, such as regular physical activity and normal weight can be seen as signs of self-regulation (Campos-Uscanga et al., 2017). And there is a growing recognition of the impact of social isolation. A recent meta-analysis of over 1,000 studies and almost 1.5 million participants found loneliness had an effect size similar to smoking and obesity in terms of the increased risk of chronic disease and mortality (Vila, 2021). For this analysis, the key features of a healthy lifestyle are regular physical activity, a healthy diet typically associated with self-regulation and some marker of psycho-social health. The key features of an unhealthy lifestyle include some marker of psycho-social stress, sedentariness and an unhealthy diet often linked with dysregulation and obesity. (Table 1)

**TABLE 1:** Features of unhealthy and healthy lifestyles.

Unhealthy lifestyle		Healthy lifestyle	
*Feature*	*Often includes one or more*	*Feature*	*Includes most of these*
**Unhealthy diet** and physically dysregulated	Smoking	**Healthy diet** and physically regulated	Non-smoker
	Excess alcohol		Moderate alcohol
	Drug addiction		No drug addiction
	Excess or under weight		Healthy weight
	Poor quality sleep, often daytime fatigue		Good quality sleep, Little daytime fatigue
**Sedentary**	Little to no routine physical activity	**Physically active**	200–400 min of aerobic activity/week
	Sedentary past-times (e.g. excess screen time)		Active past-times (e.g., gardening)
**Psychosocially distressed**	Often stressed (*Sympathetic overdrive*)	**Psychosocially stable**	Times of emotional and social well-being (*Relaxation response*)
	Little family support		Good family support
	Little social support		Good social support
	Often get dysregulated to help deal with stress		Good work/life balance

Lifestyle is a type of network in that its key features interact with each other. In an unhealthy lifestyle, for example, sustained levels of ambient stress often lead to dysregulation. Binge eating, binge drinking, and addictions are ways to seek stress-relief. When people are dysregulated, it is difficult to start and maintain a regular exercise program. With progressive sedentariness, it is easier to gain weight. In contrast, the foundational feature of a healthy lifestyle is regular physical activity (Mok et al., 2019), which has been shown to not only improve mood and decrease anxiety (Rodriguez-Ayllon et al., 2019), but also improve memory and executive functioning (Hoffmann et al., 2021)—making it easier to self-regulate and socialize.

In summary, the first three networks in this analysis include the macroenvironment of environmental and geopolitical factors, local social and cultural factors, and lifestyle itself. Lifestyle marks the pivot point between external networks and internal networks. So, what are the internal networks in the body that interact with lifestyle?

## The Immune, Autonomic and Neuro Endocrine Network

The fourth network is the mesoenvironment that mediates between the macroenvironment and microenvironment. It includes three interacting regulatory systems of the body: the immune, autonomic and neuroendocrine systems. These systems constitute a network, as there are interactions among them. So how do these interacting regulatory systems respond to a healthy and an unhealthy lifestyle?

### The Immune System

The innate immune system responds to apparent threats, be they infectious, traumatic or physiologic, and plays a central role in maintaining health. When the immune response gets dysregulated, it leads to chronic low-grade inflammation. The quintessential cell driving this process is the macrophage.

The macrophage is one of the most ubiquitous and protean cells in the body. Although best known as a white blood cell that circulates in the blood, there are also “resident macrophages” that are present in all organs and tissues. The macrophage displays multiple phenotypes or forms of cellular behaviour. In its steady state, M0 macrophages have a surveillance capacity. When exposed to hypoxia or damage-associated molecular patterns (DAMPs), macrophages change into an M1 inflammatory state and release pro-inflammatory cytokines. Cytokines are signalling molecules that are released into the microenvironment and then into the blood stream to attract cells both locally and systemically to assist in responding to the threat. Macrophages can literally engulf and consume microbes as well as cellular debris. Once the source of the threat is neutralized, macrophages go into a recuperative M2 phenotype. M2 macrophages release anti-inflammatory cytokines and assist with tissue repair and angiogenesis. Once that is complete, macrophages return to their quiescent, M0, surveillance state.

### Lifestyle and the Immune System

Food and physical activity have a profound effect on the immune system. In general, not getting enough to eat is associated with immunosuppression (Alwarawrah et al., 2018) and getting too much to eat is associated with low-grade inflammation. Food interacts with our innate immune system primarily through the gastro-intestinal tract and the microbiome. Macrophages line the intestinal tract (Chiaranunt et al., 2021). A healthy diet and a normal body mass index (BMI) are known to foster a healthy microbiome and anti-inflammatory bacteria (Sohail et al., 2019; Chen et al., 2021). Conversely an unhealthy diet high in carbohydrates and saturated fats is known to decrease diversity in the microbiome and increase pro-inflammatory bacteria (Tilg et al., 2020; Malesza et al., 2021). An unhealthy diet is associated with weight gain and the accumulation of fat cells that are filled with macrophages that produce adipokines—a type of pro-inflammatory cytokine that causes low-grade inflammation (Ouchi et al., 2011; Wynn et al., 2013).

Physical activity has both direct and indirect beneficial effects on the immune system. Skeletal muscle has been described as an immune regulatory organ as it generates a range of proteins, such as myokines, that have an anti-inflammatory effect (Duggal et al., 2019). Endurance training has been shown to increase anti-inflammatory myokines and interleukin (IL)-13 (Knudsen et al., 2020; Seaborne and Sharples, 2020). A recent meta-analysis of over 25 studies of people who were overweight or obese, exercise training decreased circulating pro-inflammatory cytokines (Gonzalo-Encabo et al., 2021). Physical activity also has an indirect effect on the immune system by fostering a healthy microbiome and this has been shown to increase immune competence (Krüger et al., 2016; Chen et al., 2021).

In contrast, sedentariness has a number of adverse effects on the immune system. Sedentariness fosters an inflammatory microbiome (O’Toole and Shiels, 2020), increases circulating pro-inflammatory cytokines (Bergens et al., 2021), and impairs the anti-inflammatory myokine response (Leal et al., 2018). The association of sedentariness and low-grade inflammation remains even when confounding factors, such as intermittent physical activity, BMI, hyperglycaemia and obesity are accounted for (Henson et al., 2013). Sarcopenia, or a progressive loss of muscle mass, strength and power, was initially linked with aging, but increasingly it is linked with sedentariness (Suzuki, 2019; Shur et al., 2021). And because sedentariness and obesity are both common, they often occur together leading to sarcopenic obesity (Batsis and Villareal, 2018), making the immune-related benefits of exercise all the more challenging to restore.

Psycho-social factors appear to largely interact indirectly with the immune system through the autonomic and the neuro-hormonal systems.

### The Autonomic Nervous System

**FIGURE 2:**
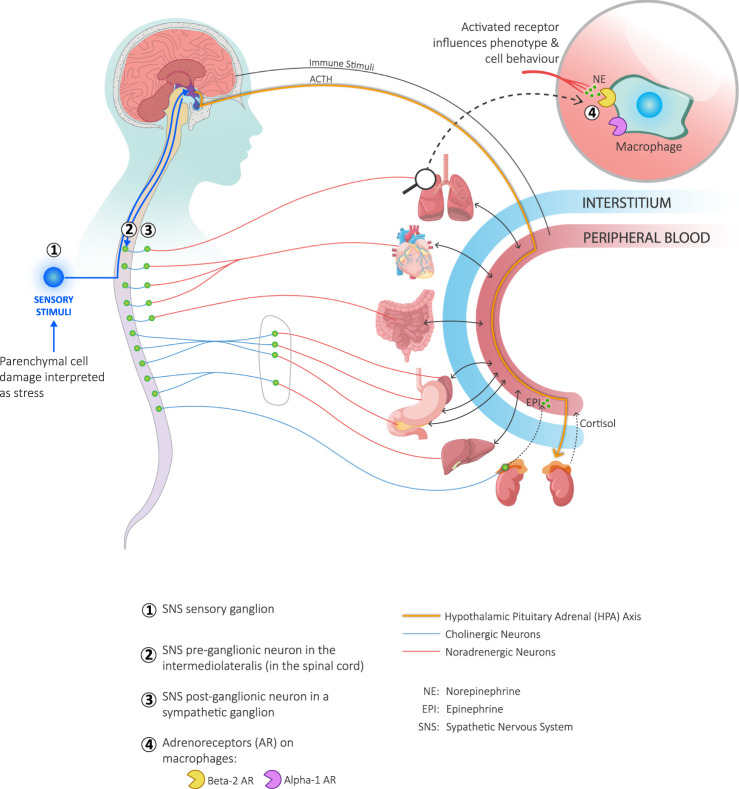
Coordination of the immune and sympathetic nervous systems with the hypothalamic-pituitary-adrenal (HPA) axis in response to parenchymal damage. Local parenchymal cell damage is interpreted as a threat by interstitial cells and as stress by sympathetic nerves. Interstitial cells respond to threats by releasing cytokines (not shown). Local sensory neurons respond to stress by activating the afferent branch of the sympathetic nervous system (SNS) and sending an impulse to the hypothalamus (lower left-hand corner of the figure). The hypothalamus responds to the information received from both the circulating cytokines and SNS stimulation (Bellinger and Lorton, 2014) in two ways: it stimulates both the efferent branch of the SNS as well as the HPA axis. Stimulation of the efferent SNS goes through the preganglionic neuron in the intermediolateral nucleus of the spinal cord (green circles). Stimulated preganglionic neurons transmit the signal through a cholinergic axon to post-ganglionic neurons (green circles) and, in a metameric fashion, through a noradrenergic axon to the affected organ where norepinephrine is released. A preganglionic neuron may also transmit directly to chromaffin cells in the adrenal medulla which in turn stimulates the release of epinephrine directly into the blood stream. When the HPA axis is stimulated, the hypothalamus releases corticotropin releasing hormone that stimulates the release of adrenocorticotrophic hormone (ACTH) from the anterior pituitary. ACTH binds to receptors on the adrenal cortex and stimulates the release of cortisol into the blood stream. Both epinephrine and norepinephrine interact with adrenergic receptors on immune cells, including macrophages (upper right-hand corner of the figure). This activates signalling pathways that constitutes one of the influences on the phenotype and behaviour of macrophages.

The autonomic nervous system (ANS) and the immune system often provide an integrated response to perceived threats (Bhowmick et al., 2009; Shouman and Benarroch, 2021). Tissue disruption will evoke both an immune cell reaction and a sympathetic response—be it from injury, infection or pathology arising in the parenchymal cells of an organ (Bellinger and Lorton, 2014). Furthermore, a sustained sympathetic response can disrupt the immune response (Bellinger and Lorton 2014; Kenney and Ganta 2014). Prolonged stress, from recurrent physical threats, anxiety, uncertainty and negative social experiences causes low-grade sympathetic overdrive (Eisenberger et al., 2017). Sympathetic neurons release an unremitting stream of norepinephrine which bind to adrenergic receptors on the surface of macrophages and amplify the release of pro-inflammatory cytokines (Bellinger and Lorton 2014). (Figure 2) In contrast, good social experiences and psycho-social support stimulate the parasympathetic and neuroendocrine systems (Haykin and Rolls, 2021), and create a buffering effect on stress and inflammation (Vila, 2021).

Regular physical activity protects against the upregulation of inflammatory cytokines and can dampen the sympathetic response (Alemasi et al., 2019). Daily exercise stimulates a parasympathetic response via the vagal nerve and cholinergic anti-inflammatory pathways. Acetylcholine released from the vagus nerve attenuates the release of pro-inflammatory cytokines (Lujan and DiCarlo, 2013; Kenney and Ganta, 2014). The vagal nerve is best known for its effect upon the heart, resulting in a lower basal heart rate and a lower heart rate during submaximal exertion (Lujan and Dicarlo, 2013). Some mind-body exercises, such as yoga and tai chi, are known for their parasympathetic effect as measured by increased heart rate variability (Zou et al., 2018). The parasympathetic nervous system only directly innervates a few organs. Most of its anti-inflammatory effects, such as in the gut, are mediated through the cholinergic anti-inflammatory pathway (Goverse et al., 2016).

The autonomic nervous system is involved in regulating feeding behaviour. Afferent vagal nerves in the gut transmit information to the hypothalamus that stimulate efferent nerves involved with the release of leptin in adipose tissue and ghrelin in the stomach (Imai and Katagiri, 2022). Diet affects the microbiota, and the microbiota and the brain communicate with each other via the enteric nervous system as well as the immune and autonomic systems (Yoo and Mazmanian, 2017; Millar et al., 2021) forming what is known as the Microbiota-Gut-Brain axis (Cryan et al., 2019). Adverse alterations of this axis lead to gut dysbiosis that has been linked to hypertension and kidney disease (Yang et al., 2018), stroke (Battaglini et al., 2020), metabolic syndrome, diabetes, obesity (Westfall et al., 2017) and Alzheimer’s disease (Megur et al., 2020). Physical activity has an influence on the Microbiota-Gut-Brain axis. For example, in people with pre-diabetes, exercise-induced alterations in the gut microbiota were found to improve glucose control and insulin sensitivity (Liu et al., 2020).

### The Neuroendocrine System

The neuroendocrine system includes the hypothalamic, pituitary and adrenal (HPA) axis, the amygdala and mediating hormones and is linked to both the ANS and the immune system through the hypothalamus. The HPA axis coordinates the neural and endocrine responses to both stress and relaxation. A healthy lifestyle is linked with relaxing activities that stimulate a parasympathetic response. Positive social interactions stimulate both a parasympathetic response and the release of oxytocin, also known as the “bonding hormone” (Jurek and Neumann, 2018). Other neurohormones that interact in this network are melatonin and dehydroepiandrosterone sulfate (DHEAS) (Luo et al., 2020; Noushad et al., 2021). Melatonin has been shown to influence immune cell phenotype (Anderson and Reiter, 2020). A recent meta-analysis of 31 studies found melatonin had significant anti-inflammatory effects (Cho et al., 2021), but this effect may be lessened with insomnia (Nicolaides et al., 2020).

When an unhealthy lifestyle activates an inflammatory response, the sympathetic nervous system (SNS) and the HPA axis are also activated. To counteract the inflammation, cortisol is released from the adrenal gland to calm the inflammation. When inflammation becomes chronic, however, there is an uncoupling of the HPA axis from the SNS. Cortisol levels drop off and this uncoupling may contribute to chronic disease progression (Kenney and Ganta, 2014; Bennett and Sturmberg, 2016).

Although cortisol is the main hormone released to moderate the response to stress, melatonin and other neurohormones are also involved (Noushad et al., 2021). Stress and binge eating increases the release of the hormone ghrelin that increases appetite, thus creating a positive feedback loop (Bremner et al., 2020). In addition, leptin is an adipocyte-derived hormone that has an effect on both the immune and metabolic systems and is associated with the inflammatory state found in obesity and metabolic syndrome (Pérez-Pérez et al., 2017).

The different regulatory systems interact through cytokines, other biosignalling molecules, and neural stimulation. So how does this signalling occur? How is perceived threat information received and responded to locally and systemically and how does this relate to chronic disease progression?

### Intercellular Crosstalk

Intercellular crosstalk is the primary method of communication between the regulatory systems of the mesoenvironment and the immune and connective tissue cells in the microenvironment. There are a plethora of signalling molecules that mediate both pro-inflammatory and anti-inflammatory activities. These signalling molecules are picked up locally and remotely and are influenced by lifestyle-stimulated changes in the gut, brain, muscle and fat.

There are a number of aspects to this process. Chemokines and DAMPs attract macrophages and other white blood cells to a site of damage or infection. This typically leads to the release of pro-inflammatory cytokines, such as TNF-alpha, IL-1, IL-6, IL-8 and IL-12. Under other stimuli, anti-inflammatory cytokines are released such as IL-4, IL-10, and IL-13. Transforming growth factor beta (TGF-beta) is essential for the development of myofibroblasts and are initially anti-inflammatory but when sustained become inflammatory (Xu et al., 2018; Koliaraki et al., 2020). The nuclear factor kappa beta (NF-κB) signalling pathway links pathogenic and cellular danger signals. The MAPK signalling pathway transports signals through the cell membrane to the nucleus of the cell (Table 2).

**TABLE 2:** Components and examples of signalling molecules involved in intracellular crosstalk.

Component	Pro-inflammatory	Anti-inflammatory
Stimuli	LPS, IFN-γ, DAMPs, epinephrine, norepinephrine	acetylcholine
Chemokines	CXCL9, CXCL10, CXCL11, CSCL16, CCL	CCL1, CCL17, CCL18, CCL22, CCL24,
Cytokines and growth factors	TNF-alpha, IL-1-alpha, IL-1-beta, IL-6, IL-8, IL-12, IL-23	IL-4, IL-10, IL-13, IL-21, TGF-β,
Transcription factors	NF-kB, STAT1, STAT5, IRF3, IRF5,	STAT3, STAT6, IRF4,

Abbreviations: CCL, chemokine C-C motif ligand, CXCL, C-X-C motif chemokine ligand; DAMPs, damage associated molecular patterns; IFN-γ, interferon gamma, IL, interleukin; IRF, interferon regulator factor; LPS, lipopolysaccharides; NF-kB, nuclear factor kappa B; STAT, signal transducer and activator of transcription; TLR, toll-like receptor; TNF-alpha, Tumor necrosis factor alpha; TGF-β, transforming growth factor-beta

Note: This does not include receptors such as Toll-like receptors or alpha and beta adrenergic receptors that facilitate intracellular cross talk, or pathways, such as the mitogen-activated protein kinase **(**MAPK) pathway that regulate the activities of transcription factors.

Crosstalk occurs not only locally among cells but also between tissues. For example, there are a number of myokines released by muscle during exercise, that circulate in the blood stream and affect the brain by enhancing brain-derived neurotrophic factor (BDNF) production. This is thought to be linked to how exercise increases mental health and executive function (Pedersen, 2019). Myokines also interact with fat tissue. With regular aerobic activity, myokines have been linked with the browning of adipose tissue and may be one of the ways that physical activity improves body composition (Leal et al., 2018). White adipose tissue generates cytokines, hormones and growth factors that target peripheral tissues, and this has been linked with the development of metabolic syndrome, although the exact mechanism is not fully understood (Masoodi et al., 2015). So where does this crosstalk actually occur? Although biosignalling molecules are transported through the blood, a lot of intercellular crosstalk occurs in the microenvironment—and this constitutes the fifth network.

## The Microenvironment Network

The fifth network is the microenvironment, which refers to the microscopic conditions of a specific part of the body. A myocardial infarction creates a microenvironment in the heart. Pancreatic cancer creates a tumor microenvironment in the pancreas. The microenvironment is part of the interstitium that includes a microscopic reticular network of extracellular matrix (ECM) which permeates all tissues and organs and provides structure and support to organ parenchymal cells. There is recent evidence that the interstitium is continuous with surrounding fascia (Cenaj et al., 2021). Once thought to be a simple and inert web-like substance, the interstitium is now known to be a complex and dynamic fluid-filled space that responds to local, systemic and external influences. It is precisely because of the local responsiveness of interstitium, that there are many different microenvironments in the body.

**FIGURE 3:**
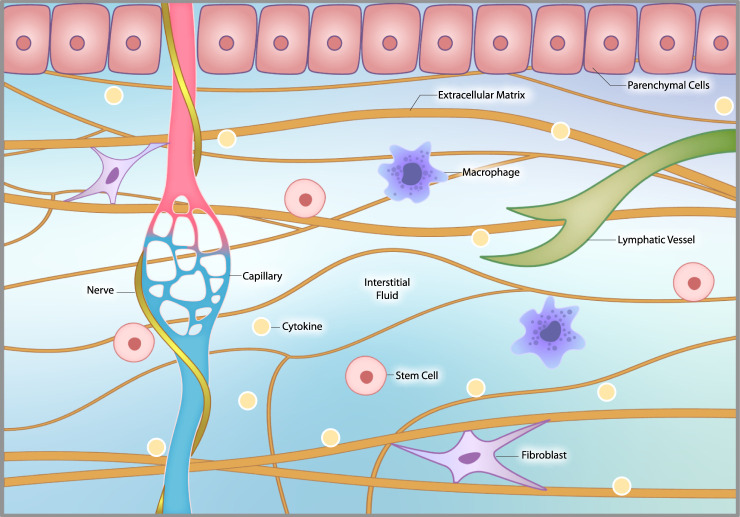
The interstitial microenvironment. The interstitium consists of extracellular matrix (ECM), interstitial fluid, interstitial cells and nerve fibers, including sympathetic nerves, that coil around local capillaries. The ECM provides structural support to organs and tissues. Interstitial fluid arises from the blood by diffusing out of capillaries. It circulates through the interstitium, interacts with interstitial cells and then is drawn into lymphatic channels and back into the venous circulation. Interstitial cells include macrophages, fibroblasts, occasional stem cells and other related cells that vary by location.

In addition to the ECM, the interstitium consists of interstitial fluid, interstitial cells, and nerve fibers (including sympathetic nerves), that coil around local capillaries (Cole et al., 2015) (Figure 3). Interstitial fluid arises from the blood by diffusing out of capillaries. (This fact is used by continuous glucose monitors that measure glucose in the interstitial fluid as a proxy for a blood glucose measurement.) This bioactive fluid circulates through the interstitium, interacts with interstitial cells and then is eventually drawn into lymphatic channels and back into the venous circulation (Scallan et al., 2010; Wiig and Swartz, 2012). Interstitial cells include macrophages and other immune cells, connective tissue cells called fibroblasts, stem cells and other cells that vary by location.

So how does lifestyle affect the interstitium and local microenvironments, and how does this affect chronic disease progression? It appears that lifestyle affects interstitial cells.

### Interstitial Cells

There are two main types of cells in the body: parenchymal cells and interstitial cells. Parenchymal cells are organ and tissue specific, such as pneumocytes in the lung, hepatocytes in the liver and myocytes in muscle. Interstitial cells are found in every organ and tissue in the body alongside the parenchymal cells and play a major role in both the normal healing process and chronic disease progression.

The two main interstitial cells are macrophages (the immune cells that have already been described) and fibroblasts. Fibroblasts are connective tissue cells that produce the ECM, which provides the scaffolding of organs and tissues. Fibroblasts also play a central role in the maintenance and repair of virtually every tissue and organ in the body, and have been described as “tutoring the immune system” (Correa-Gallegos et al., 2021). Most interstitial macrophages and fibroblasts are of fetal origin and are called resident cells. Other macrophages and fibroblasts are derived from myelopoietic stem cells in the bone marrow. These stem cells differentiate into monocytes that circulate in the blood (Clarke, 2019), and then turn into either macrophages or fibroblasts in the interstitium. Stem cells are also present in the interstitium but not all have a regenerative capacity. Stem cells in the liver can transform into hepatocytes but stem cells in the heart cannot transform into cardiomyoctyes; it is not known why (Psarras et al., 2019).

Much like cell plasticity in the brain, both macrophages and fibroblasts display tremendous plasticity. Macrophages form microglial cells in the brain, osteoclasts in bone, Kupffer cells in the liver, foam cells in atherosclerotic plaques and can also turn into fibroblasts (Haider et al., 2019). Fibroblasts can turn into stellate cells in the liver, telocytes and dendritic cells throughout the body, and differentiate into other connective tissue cells to form cartilage, tendons, ligaments and fascia (Montero et al., 2012). Fibrocytes can also revert back to stem cells (Steens et al., 2020) and become macrophages or even parenchymal cells (Inoue et al., 2019; Ieda, 2020). Fibroblasts and mesenchymal stem cells have been referred to as “two sides of the same coin” (Soundararajan and Kannan, 2018), and both can turn into myofibroblasts (Bochaton-Piallat et al., 2016) that are central to scar formation and fibrosis.

So, how does cell plasticity occur–or get impeded? What directs cells to change phenotype and their behaviour? What determines whether macrophages and fibroblasts work towards health or disease?

### The Normal Healing Process

Although the sources of injuries and chronic diseases vary widely, the body’s response to any injury or disruption is remarkably similar. Healing takes place in three consecutive phases: an inflammatory phase; a repair phase; and a final remodelling phase.

The initial inflammatory phase is an essential part of the healing process. It is typically triggered by tissue hypoxia and/or cell death. Resident and circulating macrophages changing from a quiescent M0 surveillance state into an M1 phenotype, release pro-inflammatory cytokines and this stimulates the recruitment of circulating monocytes, leukocytes and other cells. After M1 macrophages phagocytose dead cells, they present the debris to lymphatic channels for processing. Fibroblasts are also activated and release cytokines. They proliferate, migrate to the damaged tissue, and produce large amounts of ECM to isolate and repair the damaged tissue. In the case of deep wounds, myofibroblasts mobilize a prefabricated plug of interstitium-like fascia containing blood vessels, nerves, ECM and macrophages to fill and seal a wound (Correa-Gallegos et al., 2019).

In the repair phase, macrophages have a predominately M2 phenotype and release anti-inflammatory cytokines. They also support stem cell differentiation, angiogenesis, and matrix remodeling. (Chazaud, 2020; Weavers and Martin, 2020). Fibroblasts work in concert with macrophages by producing growth factors and anti-inflammatory cytokines (Psarras et al., 2019).

During the remodelling phase, fibroblasts realign the extracellular matrix with the biomechanics of the local tissue. When completed, fibroblasts return to their quiescent state, to maintain the ECM and support stem cell maintenance in specific niches (Koliaraki et al., 2020). This process has been captured on film in a mouse model of traumatic brain injury (NIH 2018; Russo et al., 2018).

### Chronic Disease Progression

Chronic low-grade inflammation occurs when this three-phase healing process gets stalled (Koliaraki et al., 2020). Specifically, when the healing process is arrested in the inflammatory phase it leads to chronic low-grade inflammation. This appears to occur when there are repetitive conditions or chronic insults that recurrently stimulate an inflammatory response (Wynn et al., 2013). This is precisely what occurs with an unhealthy lifestyle. Sedentariness, poor diet and chronic stress are recurrent stimuli for inflammatory cytokines. The effects of an unhealthy lifestyle foster the dysregulation of macrophages that then remain in an M1 phenotype. Transforming growth factor-β (TGF-β) released by lymphocytes and macrophages appears to be one of the primary factors that leads to dysregulation in fibroblasts (Wynn, 2008; Meng et al., 2016). Dysregulated fibroblast and myofibroblast activity lead to tissue fibrosis. A vicious cycle is then established as the dysregulated fibroblasts create increased tissue stiffness, which in turn stimulates dysregulated macrophages to produce more pro-inflammatory cytokines (Solis et al., 2019; Sridharan et al., 2019).

Chronic inflammation and fibrosis ultimately destroy the parenchymal tissue and leads to loss of function (Shapouri-Moghaddam et al., 2018). This specter of chronic low-grade inflammation and progressive fibrosis followed by increasing parenchymal dysfunction is remarkably similar whether it occurs in the lung (Knudsen et al., 2017), liver (Kisseleva and Brenner, 2021), heart (Frangogiannis 2019), kidney (Sulikowska et al., 2015; Meng, 2019; Lin et al., 2020), or musculo-skeletal tissue (Mahdy, 2019). Fibrosis is one of the hallmarks of end-stage chronic disease, for which there is currently no treatment.

### Heart Disease and the Microenvironment

The microenvironment in chronic disease is best understood in the heart. Unhealthy lifestyle factors activate endothelial cells lining coronary arteries to release a broad-spectrum of pro-inflammatory cytokines (Akhtar and Sharma, 2021). This attracts immune cells such as macrophages which turn into foam cells and contribute to atherosclerosis and unstable plaques (Chinetti-Gbaguidi et al., 2015). When a plaque is disrupted and blocks a coronary artery, it leads to angina and myocardial infarction.

The space between cardiac myocytes is the cardiac interstitium, which contains abundant macrophages and fibroblasts (Van Linthout et al., 2014; Pogontke et al., 2019). Hypoxic myocardial cells release DAMPs, which change macrophages from surveillance M0 phenotype to inflammatory M1 phenotype. M1 macrophages release pro-inflammatory cytokines that attract monocytes, leukocytes, and other cells to the area. (Pogontke et al., 2019; Psarras et al., 2019; Kuna et al., 2022). Monocytes mature in the damaged myocardium to create more M1 macrophages that pump out more pro-inflammatory cytokines activating fibroblasts and myofibroblasts. Fibroblasts start to pump out ECM to stabilize the injured myocardium (Haider et al., 2019). When dysregulated, myeloid cells, fibroblasts and stem cells convert to myofibroblasts, and progressive fibrosis ensues. The increasing stiffness in the myocardium and the leads to the development of heart failure (Eckhouse and Spinale 2012; Schelbert et al., 2019). Fibrosis in the heart is exacerbated when fibroblasts and stem cells adopt an osteoclastic phenotype, leading to calcified areas in cardiac tissue and atherosclerotic cardiac arteries (Psarras et al., 2019; Vergallo and Crea, 2020).

Obesity and metabolic syndrome increase sympathetic tone (Grassi et al., 2019; Bakkar et al., 2020) and can lead to the deposit of adipocytes in the heart, increasing low-grade inflammation (Berezin et al., 2020). Chronic sympathetic overdrive stimulates inflammation, and a progressive autonomic neuropathy develops in the heart associated with fibrosis. To counteract this, there have been calls for trials to treat heart failure with oxytocin to foster a parasympathetic response (Gutkowska and Jankowski 2012; Dyavanapalli, 2020; Jankowski et al., 2020).

Initial attempts to use stem cell therapies post-myocardial infarction failed. Rather than turn into cardiomyocytes, the stem cells appeared to turn into myofibroblasts and only increased the fibrosis (Packer, 2018). More recent work suggests that stem cells with a chemical inducer may help convert macrophages into a more anti-inflammatory phenotype and modify the activity of cardiac fibroblasts to make less ECM (Vagnozzi et al., 2020). Some early research suggests fibroblasts could then be induced to convert to cardiomyocytes (Cao et al., 2016).

In summary, macroenvironmental factors influence lifestyle which affect the immune, autonomic and neuro-hormonal systems in the body to influence both recovery from organ damage and chronic disease progression. Macrophages and fibroblasts in the microenvironment orchestrate the recovery process. When macrophages and fibroblasts become dysregulated, recovery stops and chronic low-grade inflammation and fibrosis begin. If perpetuated, inflammation and fibrosis lead to parenchymal damage and end-stage chronic disease. So, the final question is: How do macrophages and fibroblasts dysregulate? How does lifestyle actually change cell behaviour? This brings us to the sixth and final network.

## The Intracellular Network

It has been said: The “M1/M2 macrophage balance… governs the fate of an organ” (Shapouri-Moghaddam et al., 2018). Ultimately, chronic disease progression occurs when macrophages get stalled in an M1 phenotype and the anti-inflammatory M2 phenotype associated with healing cannot take hold. So, what determines phenotype? A number of interacting factors are involved. Addressing what is known involves delving into the intracellular network and the different types of macrophage metabolism.

Yamasaki and Eeden have asserted that macrophages phenotype and function depend primarily on the microenvironment in which they reside (Yamasaki and Eeden, 2018). Support for this includes that fact that the microenvironment reflects the neural and endocrine influences that arise from lifestyle as described above (Shapouri-Moghaddam et al., 2018; Locati et al., 2020).

Zhang and colleagues have identified that it is epigenetics, or different forms of gene expression, that determine cellular phenotype (Zhang et al., 2020). Epigenetic changes often occur in response to changes in the microenvironment. But this is not simple. For example, almost 40 different genes have been linked to M1 and M2 polarization (Murray, 2017). Locati and colleagues have discovered that it is largely one type of epigenetic change - microRNA networks - that underlie the adaptability of macrophages to different environmental cues (Locati et al., 2020). MicroRNAs can be produced locally or find their way into the microenvironment *via* the blood stream. Olivieri and colleagues have found that it is specifically the non-coding microRNAs (miRNAs), miR-21-5p and miR-146a-5p, interacting with NF-κB-driven inflammatory pathways, that mediate chronic low-grade inflammation (Olivieri et al., 2021).

But what drives the epigenetics? Díaz-Bulnes and colleagues have discovered that macrophage activation and polarization are closely linked with metabolic rewiring (Díaz-Bulnes et al., 2020). Specifically, the metabolism in macrophages changes, depending on whether they are under physiologic or stressed conditions (Peruzzotti-Jametti et al., 2021). Under physiologic conditions, cells use oxidative metabolism and the tricarboxylic acid (TCA) cycle. The proinflammatory functions of the M1 phenotype create high energy demands that overwhelm the TCA cycle so there is a switch to glycolytic metabolism (Zhu et al., 2021). This “Warburg effect” occurs in both chronic disease and cancer microenvironments—but there is a big difference between the two (Certo et al., 2021). Whereas the microenvironment in chronic disease is associated with dysfunctional M1 macrophages and inflammation, the microenvironment in cancer tissues is associated with dysfunctional M2 macrophages and immunosuppression (Hinshaw and Shevde 2019). In addition, metabolites released into the microenvironment reflect more than cell metabolism. Recent advances in metabolomics have identified that metabolites also act as master regulators of all the other “omics.” In other words, metabolites have been shown to influence gene expression (genomics), mRNA metabolism (transcriptomics), protein activity (proteomics), and cell physiology (Rinschen et al., 2019).

Ultimately, all these factors influence macrophage behaviour. Epigenetic mechanisms are turned on or off in response to changes in the microenvironment and stimulate phenotypic shifts which change the cellular metabolism within macrophages. The subsequent metabolites can then trigger new epigenetic events (Jha et al., 2015; Saha et al., 2017; Zarzour et al., 2019) that induce other changes (Zaghlool et al., 2020). The relative weight of these, and other influences, are only starting to be mapped out.

This completes the six-layered hierarchical network analysis. Level one identifies that environmental and geopolitical events can adversely affect lifestyle. Level two identifies that people are embedded in a social and cultural context that profoundly affects lifestyle. Level three considers the interactions among the different aspects of lifestyle itself. Level four explores how the regulatory systems of the body—the immune, autonomic and neurohormonal systems—respond to lifestyle factors and how they interact with each other. Level five outlines how the immune, autonomic and neuro-hormonal responses to lifestyle affect macrophages and fibroblasts in the microenvironment in ways that either help maintain or regain normal tissue function or contribute to chronic disease progression. And level six considers how the phenotype or behaviour of macrophages and fibroblasts are determined by multiple interacting factors in the microenvironment, such as cytokines, hormones, neurotransmitters, epigenetics, and metabolites. The effects of lifestyle at all these levels are summarized in Table 3.

**TABLE 3:** The features of a healthy and unhealthy lifestyle in the hierarchical six-layer network.

Network level	Healthy lifestyle		Unhealthy lifestyle
1. Macroenvironmental influences	Climatic and political stability, and no emerging infectious disease outbreaks make a healthy lifestyle possible.		Pandemics, climate emergencies and political conflicts leading to war make an unhealthy lifestyle more likely.
2. Social and cultural factors	Economic stability, access to education and health care, housing in a safe neighborhood, and being embedded in a supportive family and a healthy social network makes a healthy lifestyle more likely.		Economic instability, lack access to education ± healthcare, marginalized housing ± a dangerous neighborhood and an unsupportive family or unhealthy or absent social network makes an unhealthy lifestyle more likely.
3. Lifestyle	A diet with fresh fruits and vegetables, signs of regulation, such as a healthy weight, sound sleep, regular physical activity and support to deal with psycho-social stress constitute a healthy lifestyle.		A diet high in refined sugars and saturated fat, signs of dysregulation, such as addictions and sedentariness, as well as a lack of support for psycho-social stressors constitute an unhealthy lifestyle.
4. Immune, autonomic and neuroendocrine systems	Periodic acute inflammation and recuperation in response to injury and a balance of sympathetic and parasympathetic activity, in concert with a normal neuro-hormonal response are consistent with a healthy lifestyle.		Chronic low-grade inflammation and progressive fibrosis associated with chronic sympathetic overdrive, in concert with an abnormal neuro-hormonal response are consistent with an unhealthy lifestyle.
5. Interstitial cells in the microenvironment	Normal, regulated macrophages cycle through a surveillance phenotype (M0) that responds to injury or tissue disruption with an acute inflammatory (M1) then a reparatory (M2) phenotype.		Dysregulated M1 macrophages leads to chronic low-grade inflammation;
	Normal, regulated fibroblasts are in maintenance mode or cycling through a reactive and remodelline phenotype in response to injury, in concert with other interstitial cells.		Dysregulated fibroblasts and myofibroblasts lead to progressive fibrosis.
6. Intracellular	A healthy balance of inflammatory and anti-inflammatory cytokines, healthy neural, hormonal, epigenetic, metabolomic, and other influences, lead to well-regulated interstitial cells.		A predominance of inflammatory cytokines, and dysregulation of neural, hormonal, epigenetic, metabolomic, and other influences, lead to dysregulated interstitial cells.
	Oxidative metabolism predominates.		Glycolytic metabolism predominates (the Warburg effect).

## Discussion

This is the first time the theory has been put forward that the reason why lifestyle is linked with the primary causes of mortality is not because lifestyle affects the parenchymal cells of the heart, lungs or brain, but because it affects interstitial cells. This is based on the growing body of evidence that chronic disease progression arises from a stalled healing process orchestrated by interstitial macrophages and fibroblasts in response to a perceived threat. Network analysis was used to identify how the microenvironment, as well as social and cultural factors, influences lifestyle and how lifestyle, in turn, affects regulatory systems in the mesoenvironment and interstitial cells in the microenvironment. The analysis showed that an unhealthy lifestyle causes chronic low-grade inflammation through the dysregulation of immune cells causing chronic disease progression, and by the dysregulation of connective tissue cells causing fibrosis, that eventually interferes with parenchymal function.

### Strengths and Limitations

The strength of this theory is that it connects evidence about lifestyle with recent advances in understanding inter and intra-cellular dynamics associated with chronic disease progression. Insights into the cellular behaviour of macrophages and fibroblasts in the microenvironment have started to change how we see chronic disease and its treatment. The importance of fibrosis in chronic disease progression is only beginning to be more widely appreciated. It has recently been estimated that 40% of all deaths in the industrialized world is due to fibrosis (Friedman, 2022). And insights into interstitial cell dysregulation are starting to be incorporated into new treatment strategies. The first macrophage modifying medication has been approved for the treatment of diabetic foot ulcers (Huang et al., 2021), and a new chimeric antigen receptor (CAR) T cell is under development that uses technology from mRNA vaccines to destroy myofibroblasts in the heart, resulting in decreased fibrosis and improved myocardial function (Rurik et al., 2022). Although the microenvironment in chronic disease progression is only starting to be explored, the importance of the microenvironment in cancer is well-established. It is well-accepted that inflammatory cells are an indispensable part of cancer cell survival, proliferation, and spread (Coussens and Werb, 2002; Hinshaw and Shevde, 2019) and that this is mediated by metabolomics and epigenetics (Elia and Haigis, 2021). Several epigenetic drugs have now been authorized for cancer treatment (Bates, 2020).

The key weakness of this theory—and of network theory in general—is that it is not always clear how strong the different interactions and influences are within and between different networks. So, although important interactions between sympathetic neurotransmitters and macrophages have been identified (Bennett et al., 2018), how important these interactions are, remain unclear. The macroenvironmental influences on lifestyle have typically been underemphasized. And the relative influence of local versus circulating inflammatory cytokines remain unknown. Is there a competing effect between inflammatory adipokines from obesity and anti-inflammatory myokines from physical activity? We simply do not know. The relative strength of different influences is starting to be estimated by interactomics. A recent interactomic study of obesity found autonomic dysfunction was a hub, suggesting it has a significant influence (Geronikolou et al., 2017). Another weakness of network analysis is that it is difficult to know if all the influences and interactions have been included. For this analysis, it was clearly noted that the network described was not all-inclusive. It was silent on the effects of smoking and air pollution for example, in part because these are already well-known. There are most certainly others. Network theory lends itself to increasing complexity. The goal with this analysis was simply to identify a path from the macroenvironment through the mesoenvironment to the microenvironment; there remains much to explore.

There are other limitations to consider. First, this theory provides no insight into the actual etiology of chronic diseases. The theory explains chronic disease progression, not etiology. Why a chronic disease process takes hold in the heart, instead of the brain or the kidney is only generally addressed by the concept of repetitive insults. Second, lifestyle is an umbrella term that has been defined in multiple ways and although epidemiologic and outcome studies are compelling, what factors should be included, and the relative weights of those factors, is still being sorted out (Lohse et al., 2016). Third, our understanding of interstitial cells is still under development. It well-known, for example, that characterizing macrophage phenotypes as M0, M1 and M2 is vastly oversimplified (Murray 2017). Other transitional phenotypes have been described and completely distinct phenotypes are being discovered—such as “cloaking” macrophages that surround an injured area to protect it from an overreaction by T cells (Uderhardt et al., 2019). And there is much that remains unknown, such as when does healthy acute inflammation become unhealthy chronic inflammation (Rohleder, 2019 *)* or why does the risk of a stalled M1 phenotype tend to increase with age?

### Future Research and Clinical Implications

This theory can be tested. For example, artificial intelligence and machine learning (AI/ML) studies are designed to identify patterns arising from multiple interacting variables. Thousands of people could be followed over time who have different diets, activities and levels of psycho-social stress or well-being. Data gathered would include the usual measures of weight, food intake, activity tracking, psychosocial assessments, BMI and health outcomes, as well as heart rate variability, VO2 max, and a plethora of cytokine, genetic, epigenetic and metabolic markers. Then all this data could be analyzed by AI/ML to see what emerges. A general proof of concept for this type of study has been completed (Price et al., 2017) and more focused studies have been done, such as gathering data from thousands of people to correlate different gut microbiome compositions with different lifestyles and clinical conditions (Manor et al., 2020). AI/ML studies are limited by the quality and extent of the data used and replication of results may prove to be difficult. The results would not be definitive, but could generate new hypotheses—about the relative weight of influences for example—that can then be tested clinically.

The broader field of network analysis has confirmed that chronic disease is a consequence of perturbed interactions among multiple biological networks rather than a single altered molecular pathway. This has important implications for clinical care and future research. Network analysis has identified sub-groups within diseases, such as diabetes and Parkinson’s disease, to help explain heterogenous outcomes and tailor treatment strategies (Sturmberg, 2021; Zhang et al., 2021) and can risk stratify patients with exercise intolerance (Oldham et al., 2018). Network physiology has identified there is a direct relationship between inter-organ connectivity and survival in the critically ill (Asada et al., 2016; Tan et al., 2020). Through network physiology, we now are beginning to understand how muscle affects the immune system, fat cells, the brain and other vital organs (McGee and Hargreaves, 2019; Gomes et al., 2020). And there have been new insights into the muscle denervation process that occurs with aging and why once sarcopenia is established, it becomes increasingly challenging to stop (Pascual-Fernández, 2020; Gustaffson and Ulfhake, 2021).

The big question is: Can this knowledge be used to help people adopt a healthier lifestyle? Understanding the network of interacting factors that influence health may contribute to the new concept of whole person health that encompasses interconnecting factors across physiological, psycho-social and environmental domains (Langevin, 2022). Understanding that interstitial cells throughout your body are either working to maintain your health or are starting to dysregulate and undermine your health may enable a more proactive approach to wellness, consistent with current trends in participatory and personalized medicine (Hood and Auffray, 2013; Topol, 2019; Lamb, et al., 2022).

Perhaps most importantly, understanding that lifestyle has an effect on cell behaviour provides a physiologic basis to the re-emerging interest in salutogenesis, or the study of how health is maintained and restored (Becker and Rhynders, 2013; Langevin, 2022). Salutogenesis can be applied to prevent and mitigate chronic disease. In contrast to the predominant health care strategy that focuses on interfering with parenchymal pathology and treating each chronic disease separately, a salutogenic strategy focuses on optimizing interstitial health with the potential to prevent or mitigate multiple chronic diseases simultaneously. Optimizing lifestyle is part of a salutogenic strategy but not all of it. For example, interactomics has been used to identify what drugs might be repurposed to help regulate interstitial cells and mitigate all chronic diseases (Lee et al., 2021). And salutogenic strategies are not limited to chronic disease. For example, there is now a tantalizing possibility that spinal cord injury may not have to incur permanent damage after it was shown that a transcriptome was all that was needed to activate a latent regulatory program to remyelinate nerve cells (Monje, 2021).

## Conclusion

Chronic diseases represent a huge burden of illness for people and the health care system. As more becomes known about the hierarchy of interacting factors that influence interstitial cell behaviour, it is bound to change how chronic disease progression is understood and managed. Network physiology and network medicine can contribute to the emerging field of salutogenesis to help identify new ways to support healthy lifestyle change as well as new strategies to arrest cell dysregulation and restore health.

## Data Availability

The original contributions presented in the study are included in the article, further inquiries can be directed to the corresponding author.
